# Exploring slow-release plasma nitrogen nano-fertilizer for improved maize production and nutrient use efficiency

**DOI:** 10.3389/fpls.2026.1883384

**Published:** 2026-09-10

**Authors:** Stewart Kyebogola, Richard Onwonga, Stella Kabiri, Onesimus Semalulu, Swidiq Mugerwa, Russell Shelley Yost, Samuel Ikendi, Dennis Beesigamukama, Julius Opio, Godfrey Sseruwu

**Affiliations:** 1 Department of Land Resource Management and Agricultural Technology, Faculty of Agriculture, University of Nairobi, Nairobi, Kenya; 2 National Agricultural Research Organization, Kampala, Uganda; 3 Strategic Partnership Office, Sasakawa Africa Association, Addis Ababa, Ethiopia; 4 Department of Tropical Plant and Soil Science, University of Hawai’i at Mānoa, Honolulu, HI, United States; 5 University of California Agriculture and Natural Resources, University of California Merced, Lake Road, Merced, Merced, California, United States; 6 International Centre of Insect Physiology and Ecology, Nairobi, Kenya

**Keywords:** climate change, fertilizer, maize, nano, nitrogen, plasma, slow-release, use efficiency

## Abstract

The use of nitrogen (N) fertilizer in maize production is constrained by scarcity, low use efficiency, and high costs. Plasma technology presents a more environmentally friendly nitrogen source (PN). When incorporated into nanocellulose composites to control fertilizer release (NPN), they improve N supply and mitigate environmental risks, thereby optimizing benefits. However, the productivity of NPN relative to urea and its nanoform (NU) remains unexplored. Screenhouse and field experiments were conducted twice on maize using five fertilizer treatments: control, urea, PN, NU, and NPN. All treatments were applied once at 66 kg N ha^-1^ and replicated four times. Maize cultivated in plots treated with nano-fertilizers showed increased plant height, chlorophyll content, and stem and leaf area. Similarly, maize yield components, including kernel row number, grains per row, and 100-seed weight, were enhanced by the application of nano-fertilizers. Compared to urea, NU and NPN increased the grain yield by 46-49% and 34-85%. Compared to PN, the increases were 8-53% and 36-37%, respectively. Nano-fertilizers boosted yield by 81-235% relative to the control. Furthermore, the Nitrogen Recovery Efficiency increased to 84% and 83% for NU and NPN, respectively, from 16-57% for urea and 10-50% for PN. The uptake affected plant utilization, causing higher Agronomical Efficiency in maize with nano-fertilizer application. The high performance of nano-fertilizers, once validated through economic and on-farm evaluations, suggests that urea and PN nanoencapsulation could enable a single application to meet all N requirements for maize production. These findings indicate that the use of nano-fertilizers can reduce nutrient loss, fertilizer requirements, and the associated environmental risks.

## 1 Introduction

Low soil nitrogen levels in sub-Saharan Africa (SSA) increasingly threaten the productivity of maize (Zea mays L.) (Curtis et al., 2020; Kenawy et al., 2020; FAO, 2021; Erenstein et al., 2022; Dey and Sadhukhan, 2024; Li et al., 2024). To address this challenge, fertilizer application is a key approach to improving soil nitrogen levels for sustainable crop growth in SSA (Kyomuhendo et al., 2018; Falconnier et al., 2023). Unfortunately, mineral nitrogen fertilizers are costly and inaccessible to resource-constrained farmers, and the adoption of climate-resilient practices remains inadequate (Li et al., 2018; Fungo et al., 2019; Menegat et al., 2022; Musyoka et al., 2019; Kyebogola et al., 2024; Sabina et al., 2025). These issues collectively hinder maize productivity and threaten regional food security. Improving access to and affordability of fertilizers and soil amendments is vital for building confidence among African farmers, especially small landholders (Giller et al., 2021). Furthermore, the Abuja Declaration of the African Union (2006) suggests that increasing fertilizer application rates to 50 kg ha^-1^ in SSA could be the primary means of improving crop productivity in the region. However, many farmers still struggle to meet this target because of limited access and low fertilizer-use efficiency (Winnie et al., 2022).

Advancements in precision fertilizer application technologies can reduce fertilizer rates and costs while enhancing farm productivity (Kolapo et al., 2025). In line with the Nairobi Declaration on Africa Fertilizer and Soil Health (African Union, 2024), the document underscores the importance of initiatives that bolster local farmers’ abilities in integrated soil fertility management (ISFM) practices, domestic fertilizer production, and local supply chains, thereby fostering a collective commitment among the stakeholders. These initiatives aim to boost efficiency, resilience, and sustainability by improving soil health and customizing fertilizer use for specific soils, crops, and climatic conditions. Notably, a key action of the Nairobi Declaration was the creation of fertilizer products derived from locally available nutrient streams and innovations in local low-carbon or green ammonia production. Such efforts empower local farmers to adopt soil health-enhancing practices that increase maize yields, thereby ensuring food security and promoting the stewardship of the environment.

Although synthetic nitrogen fertilizers, such as urea, have boosted global agricultural output, their negative impacts, including resource depletion and high energy demand (0.48 MJ/mol) for production (Li et al., 2018), soil degradation, and greenhouse gas emissions, are becoming increasingly apparent (Fungo et al., 2019; Musyoka et al., 2019; Menegat et al., 2022; Sabina et al., 2025). These challenges are exacerbated by poorly timed nutrient release, leading to over 50% nitrogen loss, low (<30%) use efficiency (NUE), and stagnant crop yields (Ning et al., 2017; Mejias et al., 2021). Technologies such as plasma and nanotechnology, which facilitate the climate-smart production of slow-release fertilizers only from abundant local renewable resources of atmospheric air, water, solar energy, and plant waste, could improve their availability and encourage their adoption by farmers (Verma et al., 2023; Kyebogola et al., 2024; Manaigo et al., 2024). The plasma nitrogen (PN) fertilizer production process enhances fertilizer availability through local production, reduces transport-related costs, protects the environment, and minimizes resource waste and exploitation (Li et al., 2018). The recent use of domesticable, carbon-free nonthermal plasma technology for nitrogen fertilizer production has reduced energy requirements (0.2 MJ/mol) and could lower prices, thereby enhancing the sustainability of farming systems (Li et al., 2018). Nanoencapsulation of plasma-generated nitrogen is essential for reducing nitrogen losses, addressing significant risks, and increasing yields, as nanoparticles offer improved physicochemical properties of fertilizers and biological properties, such as a large surface area-to-volume ratio (Abdel-aziz et al., 2021). The integration of plasma and nanotechnology can harness local resources to produce slow-release nano-plasma nitrogen fertilizers (NPN), thereby enhancing the sustainable bioavailability of nitrogen, improving delivery, and boosting plant absorption efficiency. This approach has a greater multiplier effect when nitrogen fertilizer is encapsulated with affordable plant-waste-based nanocellulose to release nutrients in response to crop growth demands (Wang et al., 2021), contributing to outcomes 2 and 3 of the Nairobi Declaration of the African Fertilizer and Soil Health (AFSH) (African Union, 2024). This method could reduce dependence on the Haber-Bosch (HB) process, minimize nutrient loss and fertilizer waste, increase yield per unit area, and enhance overall efficiency with the same amount of fertilizer, thereby safeguarding the environment.

Nano-fertilizers enhance yield and NUE by gradually releasing nutrients in synchrony with plant metabolic demands. Lawrencia et al. (2021) and Rahman et al. (2021) demonstrated that nano-fertilizers with sustained-release properties optimize nitrogen uptake, improve crop growth, and minimize environmental pollution. This supports more sustainable agricultural practices by reducing resource wastage. The development of NPN can be expedited by converting liquid plasma nitrogen into crystals, followed by graft polymerization with nanocellulose sourced from local plant waste. Similarly, other efficient nano-fertilizers from urea (Chhowalla, 2017; Wang et al., 2020, 2021; Helal et al., 2023) have shown enhanced yield and NUE in grain crops through improved nitrogen absorption and assimilation (Wang et al., 2020; Kenawy et al., 2020; Helal et al., 2023). Although nano-fertilizers are often considered more effective than conventional fertilizers (Mejias et al., 2021), significant gaps remain in the evaluation of the performance of Nano Plasma N (NPN) through a comparative analysis with nano-urea (NU) and their conventional forms in maize production. Therefore, validating NPN performance would enable agronomically viable recommendations for tailoring its use to diverse conditions.

Prior research has mostly been conducted in laboratory settings, underscoring the need to assess the agronomic and physiological performance of NPN in maize compared with similar fertilizers through consistent pot experiments and field trials (EN 13266, 2001; Ozores-hampton and Carson, 2013). Such a study would establish a benchmark for measuring the effectiveness of NPN in boosting yields (Ozores-hampton and Carson, 2013; Curtis et al., 2020; Anyega et al., 2021; Godebo et al., 2021). This research sought a sustainable system for intensive crop production by investigating, for the first time, how nano-plasma nitrogen fertilizer could be used in place of plasma N and conventional inorganic nitrogen fertilizers in their nano-form for maize production. This comparison aims to guide the identification of NPN as an alternative fertilizer to increase maize yield and NUE, thereby offering insights into improving fertilizer quality and promoting environmental sustainability.

## 2 Materials and methods

### 2.1 Description of the experimental site

**Figure 1:**
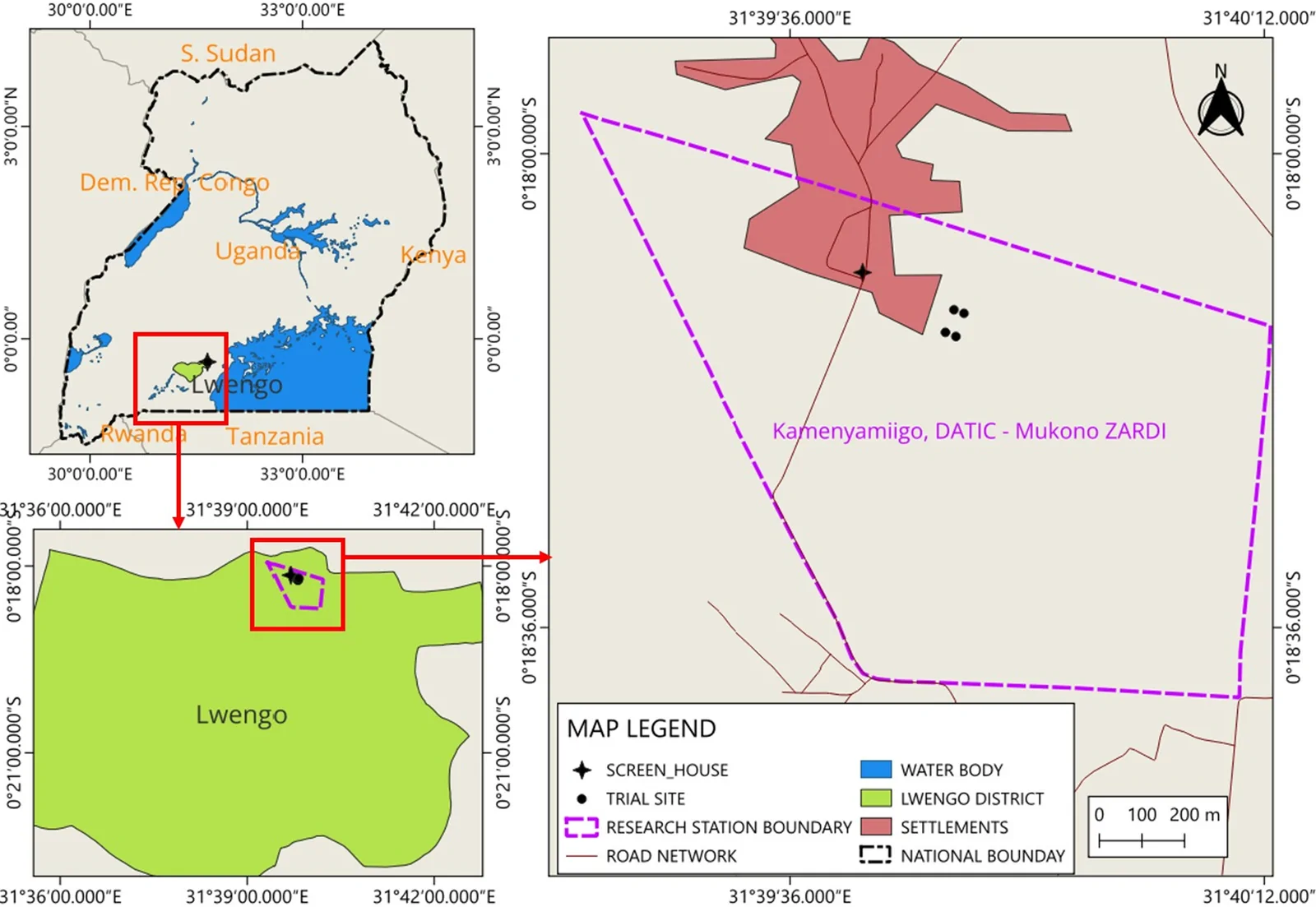
A map of Uganda showing the experiment site at Kamenyamiigo.

The study on slow-release nano-fertilizers was conducted at the Kamenyamiigo Research Station (0°18’12.1”S, 31°39’56.0”E, 1,242 m above sea level), a facility of the National Agricultural Research Organization (NARO) in the Lwengo District of Central Uganda. The soils at the study site are primarily Ferralsols (Kyebogola et al., 2020). This region falls within the Lake Victoria agroecological zone (Kyebogola et al., 2020; **Figure 1**). The climate is tropical, wet, and dry, with a bimodal rainfall pattern and an average annual rainfall of 1,350 mm (Kyebogola et al., 2020). The first rainy season spans March to May (Season A), and the second from August to November (Season B). The average temperature ranged from 16 to 30 °C.

**Figure 2:**
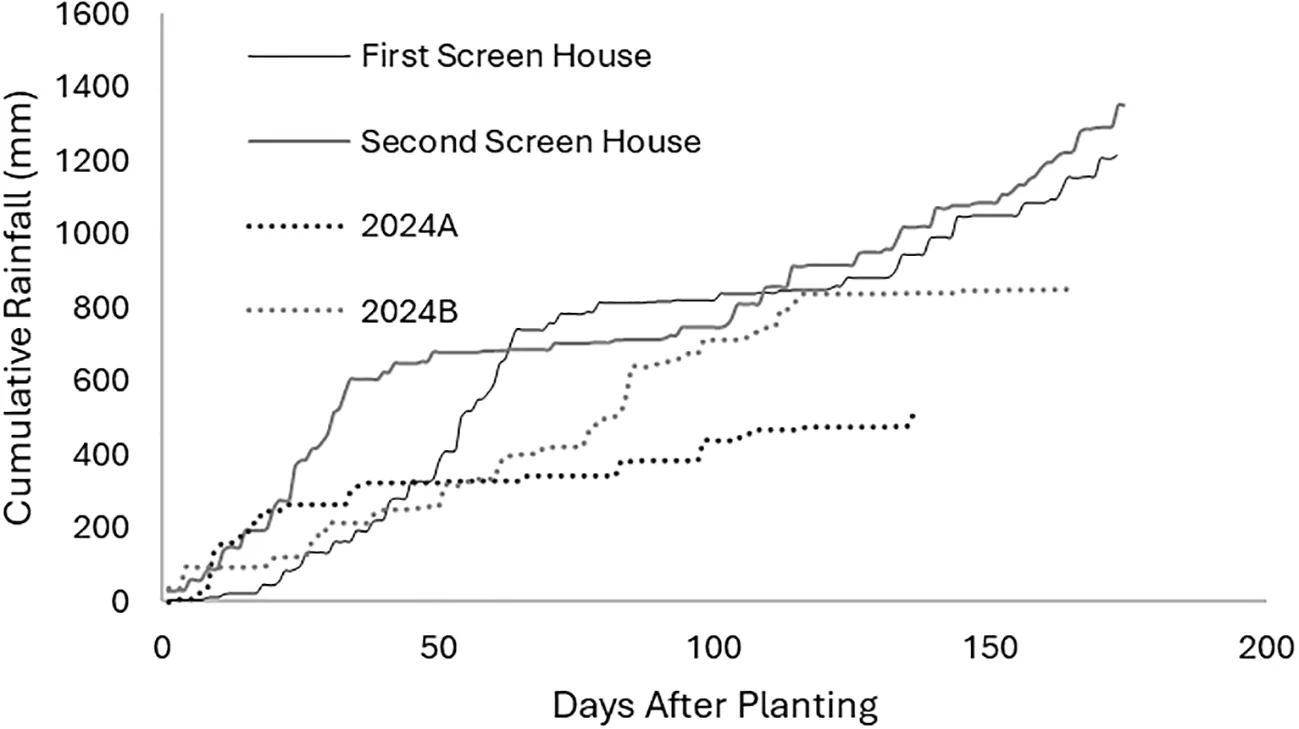
Cumulative daily rainfall during the screenhouse and field experiments at Kamenyamiigo research station.

**Figure 3:**
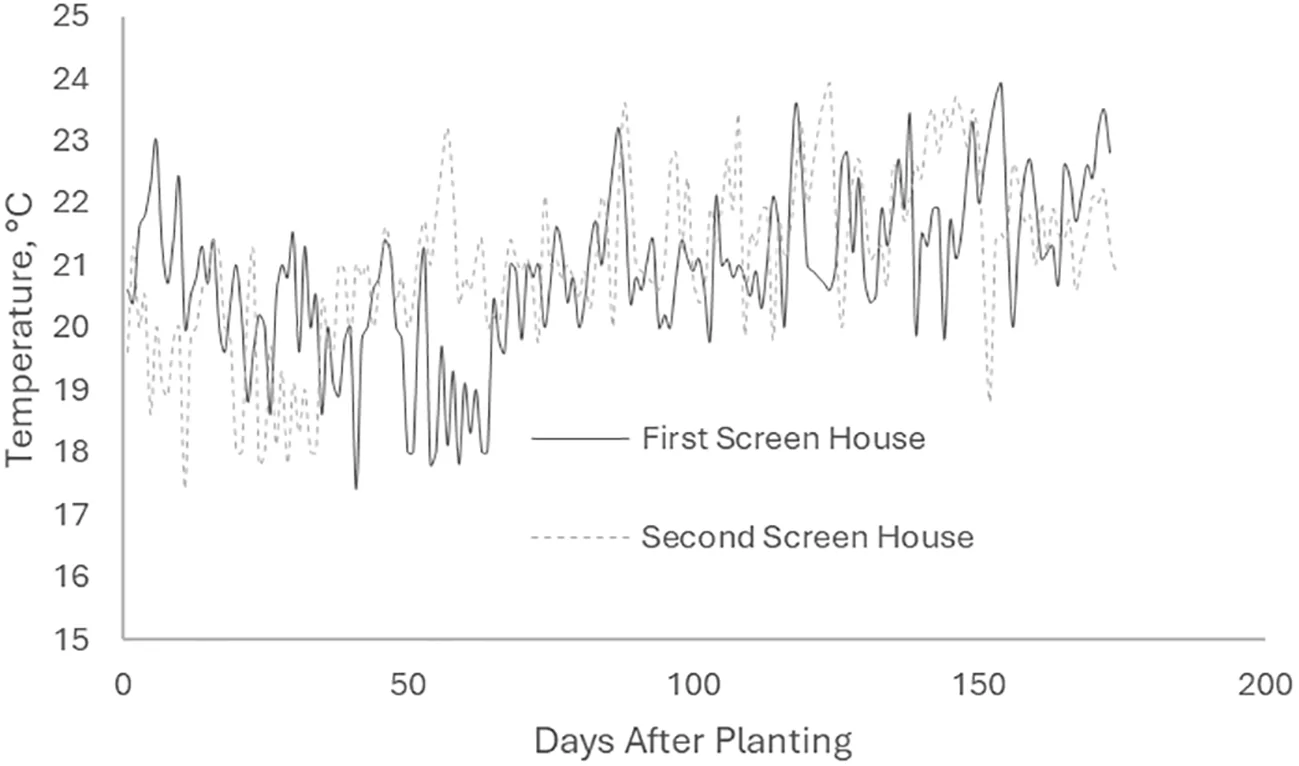
Average daily temperature during screenhouse experiments at Kamenyamiigo research station.

**Figure 4:**
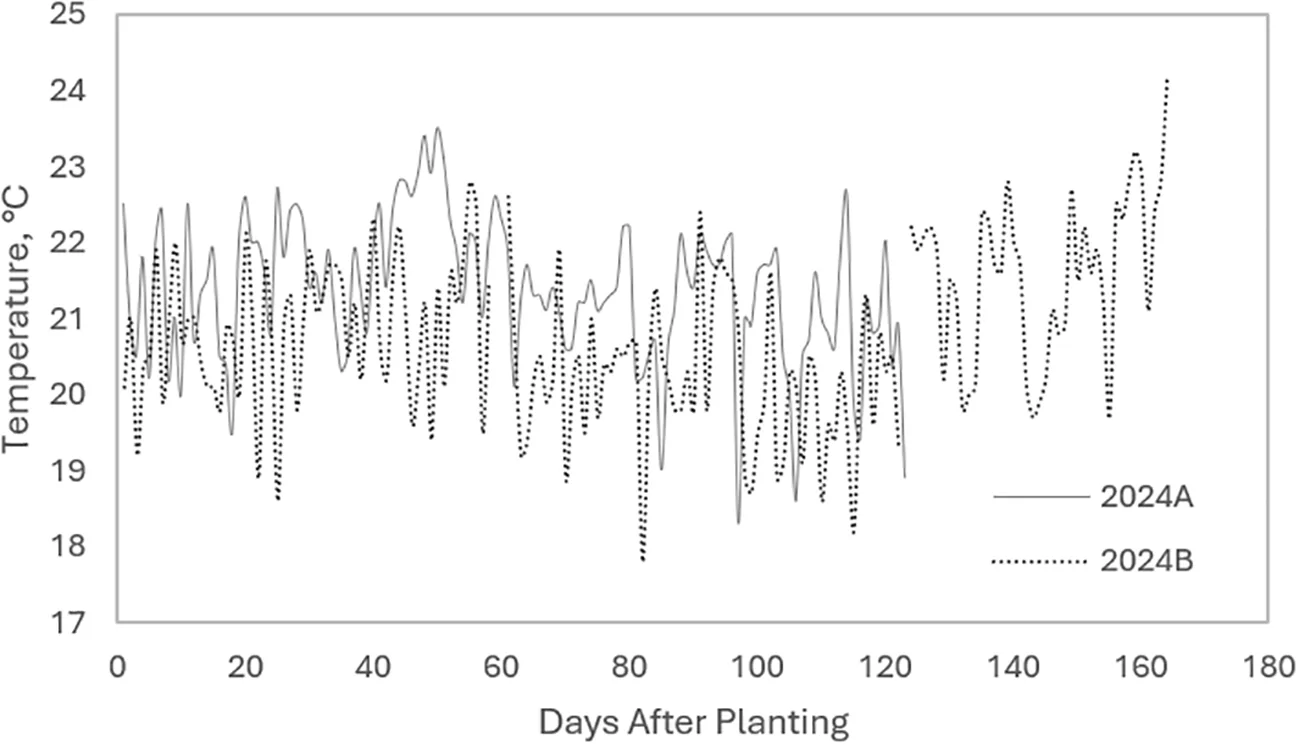
Average daily temperature during field experiments.

During the experiment, the meteorological weather station at the Kamenyamiigo farm was used to gather daily temperature and rainfall data for both the screenhouse and on-station field trials. Cumulative rainfall totals of 1,213 mm and 1,293 mm were recorded during the first and second screenhouse experiments, respectively (**Figure 2**). For the 2024A and 2024B seasons, cumulative rainfall totals of 507 mm and 849 mm were recorded, respectively (**Figure 2**). The 2024A season experienced higher average monthly rainfall over a shorter duration than the 2024B season did. The mean daily temperature for both crop cultivation periods in the screenhouse ranged from 17.4 to 23.9 °C (**Figure 3**). However, during the 2024A season, the mean daily temperature varied from 18.3 °C to 23.5 °C, whereas in the 2024B season, it ranged from 17.8 °C to 24.2 °C (**Figure 4**). Overall, the average temperature was higher during the 2024A season than during the 2024B season.

### 2.2 Sources of fertilizers

The experiment utilized four types of fertilizers: locally available commercial urea (46% N), plasma nitrogen (PN) (1% N), and a liquid nitrate fertilizer. The PN was produced using plasma technology in a laboratory in Kamenyamiggo, Uganda. Plasma nitrogen (PN) is generated by introducing atmospheric air into a solar-powered plasma reactor, producing nitrous oxide, which is then stabilized with water to form a liquid nitrate (Kyebogola et al., 2024). Nano plasma N (NPN), containing 15% N, and nano urea (NU), containing 26% N, were developed through *in situ* graft solution polymerization of urea or a PN-based salt of ammonium chloride on cellulose nanoparticles derived from banana peels (Lohmousavi et al., 2020).

Briefly, PN was first converted to ammonium chloride salt via electrocatalysis (Liu et al., 2023a). This process increases the N concentration in the fertilizer, enabling easier application. Subsequently, the salt fertilizer was graft polymerized onto nanocellulose (BPCNF) mechanically extracted from banana peels (Lohmousavi et al., 2020). This was achieved by melting 90 g of fertilizer in a beaker on a hot plate at 80 °C for 3 min. Then, 25 g of BPCNF was added to the fertilizer, and the mixture was stirred for 5 min to ensure thorough mixing. Subsequently, 10 g of PVA was added to a conical flask containing 200 ml of distilled water along with 10 g of BPCNF. The mixture was heated to 80 °C for 15 min under magnetic stirring, resulting in gel formation. This gel was added to the BPCNF-fertilizer mixture and stirred uniformly. After cooling, acetic acid (AA) was added to the mixture, which was then stirred. The mixture was then poured into a mold and extruded into a pelletized slow-release fertilizer with an 8 mm diameter, called NPN. Subsequently, the NPN was dried at 50 °C for 12 h before storage and application. A similar process was followed to formulate nano-urea from urea fertilizer.

### 2.3 Experimental setup and design

This study included both screenhouse and field experiments. The screenhouse experiment aimed to compare responses under semi-controlled conditions, bridging the gap between laboratory and open field conditions. The field experiment aimed to test the treatments under real, uncontrolled farm conditions.

#### 2.3.1 Screenhouse experimental setup

The screenhouse experiment was conducted over two cycles between September 2023 and April 2024. The soil for the screenhouse experiment was collected from a depth of 0–30 cm in July 2023, precisely at the location where field trials were conducted during the 2024A and 2024B seasons. A sample was collected for soil composition analysis prior to the experiment. Prior to these seasons, the site was used for maize cultivation. However, the predominant vegetation at the trial sites consisted of blackjack (***Bidens pilosa***) and nut grass (***Cyperus rotundus***), which are commonly found in areas with low soil fertility (Kyebogola et al., 2020).

The screenhouse experiment used two plastic pots, each measuring 30 × 45 cm, which were nested together and separated by a ¾-inch spacer. The inner pot featured screened drain holes, whereas the outer pot was equipped with a fixed tap at its base to drain the leachate and collect it for additional leaching studies. Each inner pot was filled with 10 kg of soil and watered until saturation. The study included five treatments: control (no nitrogen fertilizer applied), Urea, Plasma N, nano-urea (NU), and Nano Plasma N (NPN). The treatments were randomly assigned to pots using a completely randomized block design (CRBD) with four replicates. The top 5.0 cm of soil in each pot received a known amount of nitrogen fertilizer (1.44 g N), corresponding to the recommended rate of 66 kg N ha^-1^ for maize production in Uganda. For the PN treatments, 148.5 ml of PN was applied per plant by drenching. Nevertheless, the water volume of PN was not equalized across other treatments. Phosphorus and potassium were applied at blanket rates as triple superphosphate (TSP) and muriate of potash (MOP), respectively, based on soil analysis, to eliminate macronutrient limitations to nitrogen uptake, in accordance with Uganda’s maize production guidelines (MAAIF, 2019).

Maize was chosen as the test crop because of its high fertilizer nutrient requirements and importance as both a food and cash crop in Uganda (Ikendi et al., 2024). The variety selected for this study was Bazooka, which is known for its high yield, compatibility with intercropping with other common crops such as beans, and resistance to maize lethal necrosis, a condition that can lead to complete maize loss, particularly during droughts (MAAIF, 2019). Two seeds were planted in each pot, with 5 liters of water provided at planting. After germination (i.e., 10 days after planting), one seedling was retained in each pot. This arrangement was used for the first screenhouse experiment, which began on September 25, 2023, and the second experiment commenced exactly 30 days later.

#### 2.3.2 On-station field trial setup

Prior to setting up each field trial, two soil samples were taken from depths of 0–30 cm and 31–50 cm. These samples were packed and stored in an ice-cooled box before being transported to Makerere University in Kampala for laboratory analysis. The soil samples were then analyzed according to the standard laboratory procedures outlined by Soil4Africa D3.4 (2021). Accordingly, these soils exhibited low pH, organic carbon, nitrogen, phosphorus, and cation exchange capacity (CEC) levels. The soil had a sandy clay texture (**Table 1**), and the exchangeable bases were generally above the critical level.

**Table 1:** Selected physicochemical properties of the soil used for the experimental study.

Experiments	Soil depth (cm)	pH	OC	SOM	N	P	CEC	Exc. Al	Ca	Mg	K	Na	Sand	Silt	Clay	Texture
			‘-–%-–			ppm	‘-––cmol (+)/kg soil-––-						‘-––-%-––			
Screenhouse 1 and 2	0-30	5.8	1.6	2.7	0.11	2.1	7.9	0.10	4.4	1.3	0.1	0.05	50.8	8.0	41.2	Sandy clay
2024A	0-30	5.7	2.3	4.1	0.14	8.8	12.3	0.10	6.0	1.8	0.7	0.07	52.3	11.2	36.5	Sandy clay
	31-50	4.8	1.7	3.0	0.12	7.0	14.1	0.14	3.9	1.8	0.2	0.07	48.0	6.0	46.0	Sandy clay
2024B	0-30	6.2	2.0	3.5	0.24	1.2	16.9	0.0	9.2	6.0	0.8	0.01	52.0	12.0	36.0	Sandy clay
	31-50	4.4	1.4	2.4	0.17	0.4	10.6	0.86	5.2	4.0	0.7	0.01	48.0	6.0	46.0	Sandy clay
Critical value*		5.5	N/A	3	0.25	15	16	0.1	4.50	0.20	0.22	N/A	N/A	N/A	N/A	

*Source of critical values denoted (Beesigamukama et al., 2020). Key: N/A, not available.

Each season, the trial site was plowed once with a tractor two weeks prior to planting. Field trials were conducted from April to August 2024 (2024A season) and from August 2024 to February 2025 (2024B season). The trials were conducted on plots measuring 6.0 m × 9.0 m, with a 2.0 m gap between plots. Each fertilizer treatment (control, i.e., no N fertilizer added; urea; PN; NU; and NPN) was replicated four times, resulting in 20 experimental plots. Field trials were conducted using a completely randomized block design (CRBD).

Fertilizer was applied to planting holes spaced 75 cm × 60 cm apart within the treatment plots (nine rows and 144 planting holes per plot). The application rate followed those applied in a screenhouse. Blanket rates of P and K were applied before the holes were half-filled with soil, specifically 27 kg P ha^-1^ as Triple Superphosphate (TSP) and 57.2 kg K ha^-1^ as Muriate of Potash (MOP) (MAAIF, 2019). Subsequently, three seeds of the Bazooka maize variety (UH 5354) were placed in each planting hole, and the holes were filled with soil. Following germination, the seedlings were thinned to two plants per hill (288 plants per plot).

### 2.4 Management of experimental trials

In accordance with standard agronomic practices, the treatment plots were maintained weed-free through three rounds of weeding and the manual removal of any newly emerged patches. Fall armyworms were managed by applying cypermethrin pesticide twice every 10 days, beginning 14 days after planting. Additionally, supplemental irrigation was provided twice per week (approximately 160 liters per plot per week) from 35 days after planting (DAP) until maize reached maturity, mitigating the effects of a prolonged dry spell due to late planting in the 2024A season. Water was delivered using a sprinkler-irrigation system. In the 2024B season, the maize garden received water twice a week during the first 30 days after planting (DAP).

### 2.5 Determination of crop growth and grain yield

Following planting in both the screenhouse and field trials, various maize growth and yield characteristics were measured, including plant height, chlorophyll content, stem girth, and leaf area (LA). In the screenhouse, measurements were taken biweekly from each treatment plant in a pot. In contrast, for the field experiments, five middle rows were selected for the data collection. From each row, two representative plants (10 plants per plot) were randomly selected and tagged for continuous data collection throughout the experiment, until harvest. Maize growth data were collected at 35, 55, 70, and 90 DAP, which corresponded to the early vegetative, late vegetative, tasseling, and silking stages, respectively, of maize growth. Plant height was measured using a tape measure from the ground to the tip of the topmost leaf of the plant. Chlorophyll content was determined using a chlorophyll meter in SPAD units, with the fully open fourth leaf from the top (Chen et al., 2023) being selected for each tagged plant. SPAD readings were taken at three positions on each leaf: near the apex, center, and adjacent to the base, and the average chlorophyll SPAD values for each plant were recorded.

Stem girth was measured using a digital Vernier caliper at the base of each maize plant, and the readings were recorded. Leaf area was determined by measuring the length (L) and width (W) of the fully open third or fourth leaf from the top of the tagged maize plants using a ruler. The values obtained were calculated using Equation 1, as outlined by Butnan and Toomsan (2019).

(1) LA=LxWx0.75

Once the plants reached maturity (approximately 140 days after planting [DAP]), they were cut at the base in a screenhouse and weighed. The yield parameters assessed included the number of kernel rows, kernel/grain per row, 100-seed weight, plant biomass, grain yield, and harvest index (HI), which were measured using standard procedures. The harvests were conducted at 173 and 174 days after planting (DAP) for the first and second screenhouse experiments, respectively. In the field experiments, harvesting was performed at 140 and 163 DAP for the 2024A and 2024B seasons, respectively. In field trials, plants in the five middle rows were counted (n = 80 plants) before harvest. Harvesting involved cutting the plants at the base, and the stover mass was measured using a digital scale. The maize ears were then removed from the harvested plants and dried. The number of kernel rows per maize cob was counted and recorded for ten cobs. In addition, the number of grains per kernel row was counted, and the average number of kernel per cob or ear was recorded for each plot. The maize grains were then hand-threshed from the cobs, and the weight per harvested plot area was determined using a scale to weigh the grains. Plant biomass and grain samples were oven-dried at 60 °C for 72 h to ensure complete drying without compromising nutritional quality. After cooling, the weights were recorded on a dry basis at a moisture content of 12.5%. Using the moisture values, maize stover and grain yields were determined on a per-plot and per-hectare basis (t ha^-1^). From each treatment plot, 100 seeds were randomly selected and weighed individually. The harvest index (HI) was calculated as the ratio of the dry grain weight to the total plant biomass. Subsamples of dry stover and maize grains were analyzed in the laboratory for total nitrogen.

### 2.6 Nitrogen use efficiency determination

The total N uptake measured in plant tissues and grains was used to determine various NUE parameters. Physiological Efficiency (PE) evaluates the yield gain per unit of fertilizer nutrient absorbed by the plant, whereas Agronomic Efficiency (AE) measures the economic yield gain per quantity of nutrients applied. Agrophysiological Efficiency (APE) was computed as a measure of the economic yield gain per nutrient absorbed by the plant, whereas the Nitrogen Harvest Index (NHI) served as a measure of the fertilizer nutrient utilized by the grain compared to the nitrogen absorbed by the plant. The recovery efficiency (RE) assesses the ability of plants to acquire nutrients from the soil (Godebo et al., 2021; Randhawa et al., 2021; Li et al., 2024). Equations 2-6 were used to calculate NUE.

(2) $$\text{RE}=\frac{(\text{Nutrient uptake F},\text{ kg }-\text{Nutrient uptake C},\text{ kg})\;}{\left(\text{Quantity of nutrients applied},\text{ kg}\right)}\text{ x }100=\%$$

(3) $$\text{PE}=\frac{(\text{Yield F},\text{ kg }-\text{Yield C},\text{ kg})\;}{\left(\text{Nutrient uptake F},\text{ kg}-\text{Nutrient uptake C},\text{ kg}\right)}={\text{kg kg}}^{-1}$$

(4) $$\text{APE}=\frac{(\text{Yield F},\text{ kg }-\text{Yield C},\text{ kg})\;}{\underset{\text{Straw}+\text{Grain}}{(\text{Nutrient uptake F},\;}\text{kg})\;-\underset{\text{Straw}+\text{Grain}}{(\text{Nutrient uptake C},\;}\text{kg}))}={\text{kg kg}}^{-1}$$

(5) $$\text{AE}=\frac{(\text{Yield F},\text{ kg }-\text{Yield C},\text{ kg})\;}{\left(\text{Quantity of nutrients applied},\text{ kg}\right)}={\text{kg kg}}^{-1}$$

(6) $$\text{NHI}=\frac{(\text{N in maize grain},\text{ kg})\;}{\left(\text{Total N content in plant},\text{ kg}\right)}\text{x }100=\%$$

### 2.7 Laboratory analysis of soil and plant samples

Soil samples collected for site characterization and routine analysis were initially air-dried for a week and then oven-dried at 60 °C for 12 h. The samples were then analyzed for pH, organic carbon (OC), nitrogen (N), phosphorus (P), potassium (K), calcium (Ca), and magnesium (Mg) using the standard methods outlined by Soil4Africa D3.4 (2021). The detailed results are listed in **Table 1**. Soil pH (in H_2_O) was measured potentiometrically using a 2.5:1 water-to-soil suspension ratio. The Walkley and Black wet oxidation methods were used to determine the soil organic carbon (Soil4Africa D3.4, 2021). Total soil nitrogen was assessed using Kjeldahl digestion (Soil4Africa D3.4, 2021). Available phosphorus was measured using the Mehlich 3 solution (Soil4Africa D3.4, 2021). An atomic absorption spectrophotometer was used to analyze potassium, calcium, and magnesium after extraction with ammonium acetate. Soil texture was determined using the hydrometer method, as described by Soil4Africa D3.4 (2021).

Before analysis, the dried maize stover subsamples were finely ground and sieved through a 2-mm mesh to obtain a fine powder. Subsequently, the samples were analyzed for total N using the Kjeldahl method (Beesigamukama et al., 2020).

### 2.8 Statistical analysis

Data collected from field experiments on plant height, stem girth, chlorophyll content, and leaf area showed significant variations across the different crop growth stages. Consequently, datasets from both the screenhouse and open-field experiments were analyzed according to maize growth stage. To assess the factors directly influencing crop productivity, maize yield data, including kernel rows number, grains per kernel row, grain yield, 100-seed weight, and HI, were examined. Box plots were generated for the grain-yield data for each season. Nutrient-use efficiencies were analyzed using the same statistical procedures. Owing to seasonal differences, the variables were analyzed separately for each season.

The impact of the fertilizers on maize plant growth, yield characteristics, and NUE was assessed using one-way analysis of variance (ANOVA) within a completely randomized block design (CRBD) using Genstat 11.1. Prior to analysis, the data were evaluated for normality and homogeneity of variance to ensure the validity of the statistical tests. *Cook’s* statistics (Payne et al., 2009) were used for this analysis. When significant differences were detected, Fisher’s protected Least Significant Difference (LSD) at a 0.05 probability level was used to compare means.

## 3 Results

### 3.1 Effects of urea, PN, and their nano-fertilizer forms on maize growth characteristics in screenhouse and field trials

The application of fertilizer treatments in the initial screenhouse experiment resulted in significant variations in maize plant height at 84 days after planting (DAP). Notably, plant height reached its maximum at 84 DAP, with the control and NPN treatments yielding the shortest and tallest plants, respectively. The NPN treatment produced significantly taller plants than the NU treatment and control groups (**Table 2**). Furthermore, maize plants treated with urea and PN were significantly taller than those in the control group. In the second screenhouse experiment, fertilizer application significantly influenced plant height at 56, 70, and 84 days after planting (DAP). Specifically, at 56 and 84 DAP, the NPN treatment resulted in a significant increase in plant height of 12–29 cm compared with the other treatments. During the 2024A and 2024B seasons, on-station trials revealed no significant effects of fertilizer application on the plant height. However, plants treated with the nano-fertilizer consistently exhibited greater height than those treated with other fertilizers throughout the 2024A season (**Table 3**). Additionally, plants in plots treated with the nano-fertilizer attained their peak height at 70 DAP, whereas the other treatments peaked at 90 DAP in both the 2024A and 2024B seasons. In contrast, during the 2024B season, plants from plots treated with nano-fertilizer were shorter than those in the control, PN, and urea-treated plots at all growth stages.

**Table 2:** Comparative effects of urea and PN and their nano-fertilizer forms on maize growth characteristics in on-station field trials.

Parameter	Fertilizer	First screenhouse						Second screenhouse					
		14DAP	28DAP	42DAP	56DAP	70DAP	84DAP	14DAP	28DAP	42DAP	56DAP	70DAP	84DAP
Plant height, cm	Control	25.7 ± 3.7	32.6 ± 1.3	64.9 ± 8.9	92.9 ± 8.9	110.0 ± 3.2	126.1 ± 6.9^c^	21.9 ± 5.0	55.2 ± 3.3	99.1 ± 3.3	145.7 ± 8.3^ab^	174.8 ± 8.5^b^	178.3 ± 8.7^b^
	Urea	36 ± 5.0	58.2 ± 7.0	83.3 ± 7.4	111.1 ± 7.4	125.2 ± 5.8	152.1 ± 5.2^ab^	27.6 ± 3.2	60.8 ± 6.0	96.8 ± 6.0	135.2 ± 5.7^b^	162.2 ± 5.8^c^	165.5 ± 6.0^c^
	PN	33.7 ± 2.2	53.7 ± 7.3	78.3 ± 11.3	107.1 ± 11.3	123.9 ± 10.6	152.9 ± 8.5^ab^	30.9 ± 3.0	63.6 ± 2.7	100.3 ± 2.7	139.1 ± 2.8^b^	166.9 ± 2.9^c^	170.3 ± 2.9^bc^
	NU	19.6 ± 4.9	46.5 ± 4.2	70.3 ± 5.7	103.0 ± 5.7	128.5 ± 7.5	143.7 ± 4.2^bc^	32.1 ± 1.2	70.4 ± 1.7	113 ± 1.7	146.9 ± 3.2^ab^	176.3 ± 3.2^b^	179.8 ± 3.3^b^
	NPN	30.5 ± 5.6	51.2 ± 7.2	76.4 ± 7.3	107.2 ± 7.3	132.0 ± 6.3	173 ± 12.9^a^	28.2 ± 3.5	64.1 ± 4.8	104.1 ± 4.8	158.9 ± 3.5^a^	190.7 ± 3.5^a^	194.5 ± 3.6^a^
	p value	ns	ns	ns	ns	ns	0.015	ns	ns	ns	0.046	0.034	0.042
Stem girth, mm	Control	3.4 ± 0.51	5.3 ± 1.1	7.6 ± 0.74	7.4 ± 0.74	7.4 ± 0.43	6.4 ± 0.38	4.0 ± 0.68	5.4 ± 0.84	11.2 ± 0.84^b^	15.0 ± 1.0^bc^	18.0 ± 1.1^bc^	18.4 ± 1.1^b^
	Urea	4.4 ± 0.51	7.2 ± 1.8	9.0 ± 0.79	9.3 ± 0.79	9.2 ± 0.65	8.6 ± 0.59	4.9 ± 0.45	6.45 ± 0.93	12.1 ± 0.93^b^	15.1 ± 0.60^bc^	18.1 ± 0.61^bc^	18.5 ± 0.63^b^
	PN	4.4 ± 0.26	8.3 ± 1.3	9.1 ± 0.61	8.8 ± 0.61	8.4 ± 1.1	7.6 ± 0.59	4.8 ± 0.25	6.1 ± 0.61	10.7 ± 0.61^b^	13.3 ± 0.35^c^	15.9 ± 0.36^c^	16.3 ± 0.37^c^
	NU	2.9 ± 0.25	6.9 ± 1.2	8.1 ± 0.29	8.7 ± 0.29	9.0 ± 0.52	8.0 ± 0.44	5.3 ± 0.48	6.3 ± 0.56	14.6 ± 0.56^a^	16.5 ± 1.3^ab^	19.7 ± 1.3^ab^	20.1 ± 1.3^ab^
	NPN	4.2 ± 0.41	4.5 ± 0.29	9.3 ± 0.94	8.5 ± 0.94	9.3 ± 0.60	8.2 ± 0.65	5.0 ± 0.41	5.8 ± 0.48	12.0 ± 0.48^b^	17.9 ± 0.4^a^	21.5 ± 0.41^a^	21.9 ± 0.42^a^
	p value	ns	ns	ns	ns	ns	ns	ns	ns	0.015	0.014	0.028	0.036
Chlorophyll, SPAD	Control	1.5 ± 0.24	2.3 ± 0.95	4.0 ± 1.3	7.2 ± 1.3	10.0 ± 1.4^bc^	9.7 ± 0.51	1.3 ± 0.13	3.7 ± 1.5	14.3 ± 1.5^a^	13.6 ± 1.3^bc^	16.3 ± 1.3^bc^	16.7 ± 1.3^bc^
	Urea	1.8 ± 0.10	4.5 ± 1.4	6.1 ± 0.96	9.0 ± 0.96	7.8 ± 1.1^c^	9.3 ± 2.2	1.7 ± 0.45	7.7 ± 2.4	17.1 ± 2.4^b^	12.9 ± 0.94^bc^	15.5 ± 0.96^bc^	15.8 ± 0.99^bc^
	PN	2.5 ± 0.48	4.3 ± 1.6	4.4 ± 0.61	6.8 ± 0.61	7.9 ± 0.66^c^	9.2 ± 2.2	2.3 ± 1.1	6.3 ± 0.83	12.7 ± 0.83^b^	11.0 ± 0.91^c^	13.2 ± 0.93^bc^	13.5 ± 0.95^c^
	NU	1.6 ± 0.27	2.0 ± 0.37	3.6 ± 0.96	9.1 ± 0.98	11.2 ± 0.32^ab^	8.1 ± 1.4	1.6 ± 0.26	9.0 ± 1.8	20.9 ± 1.8^ab^	16.9 ± 2.2^b^	20.2 ± 2.3^b^	20.6 ± 2.3^b^
	NPN	1.7 ± 0.33	3.6 ± 0.92	5.8 ± 1.2	10.6 ± 1.2	14.0 ± 1.1^a^	9.6 ± 1.1	2.4 ± 0.62	7.8 ± 1.1	16.2 ± 1.1^ab^	23.3 ± 1.9^a^	28.0 ± 2.0^a^	28.5 ± 2.0^a^
	p value	ns	ns	ns	ns	0.003	ns	ns	ns	0.028	0.001	0.003	0.012
LA, cm^2^	Control	18.4 ± 7.4	69.1 ± 31.1	115.0 ± 47.6	217.0 ± 47.6	263.0 ± 12.1	298.0 ± 21.0	21.8 ± 7.0	109.1 ± 34.2	262.0 ± 34.2	596.0 ± 48.9	715.0 ± 49.9	730.0 ± 51.3
	Urea	21.3 ± 1.4	68.3 ± 15.0	160.0 ± 25.4	265.0 ± 25.4	305.0 ± 19.0	313.0 ± 7.7	25.7 ± 1.7	155.6 ± 68.4	273.0 ± 68.4	543.0 ± 58.3	652.0 ± 59.5	665.0 ± 61.2
	PN	18.5 ± 0.91	126.2 ± 33.3	159.0 ± 33.7	265.0 ± 33.7	309.0 ± 41.6	319.0 ± 45.6	22.9 ± 7.0	152.7 ± 26.5	247.0 ± 26.5	539.0 ± 12.2	647.0 ± 12.4	660.0 ± 12.8
	NU	32.6 ± 4.7	78.9 ± 11.0	147.0 ± 17.9	235.0 ± 17.9	335.0 ± 30.6	348.0 ± 38.0	35.5 ± 4.5	200.0 ± 34.9	380.0 ± 34.9	591.0 ± 62.8	709.0 ± 64.0	723.0 ± 65.8
	NPN	24.2 ± 5.4	43.9 ± 7.7	159.0 ± 24.9	252.0 ± 24.9	334.0 ± 18.1	325.0 ± 19.3	28.4 ± 5.5	139.9 ± 16.8	267.0 ± 16.9	577.0 ± 28.8	692.0 ± 29.4	706.0 ± 30.3
	p value	ns	ns	ns	ns	ns	ns	ns	ns	ns	ns	ns	ns

DAP, days after planting; control, unfertilized; PN, plasma nitrogen; NU, nano urea; NPN, nano plasma nitrogen; LA, leaf area; ns, not significant. Within the same column and per parameter, means (± SE) followed by the same letters are not significantly different at p ≤ 0.05.

**Table 3:** Comparative effects of urea and PN and their nano-fertilizer forms on maize growth characteristics in on-station field trials.

Parameter	Fertilizer	35DAP	2024A season		90DAP	35DAP	2024B season		90DAP
			55DAP	70DAP			55DAP	70DAP	
Plant height, cm	Control	91.8 ± 5.2	161.8 ± 13.3	194.9 ± 13.7	205.6 ± 10.3	73.5 ± 4.1	146.8 ± 7.4	217.4 ± 20.2	228.1 ± 8.0
	Urea	95.1 ± 2.0	169.4 ± 1.5	192.1 ± 5.6	202.6 ± 7.5	74 ± 3.3	144.4 ± 3.6	230.1 ± 13.3	240.6 ± 7.3
	PN	94 ± 5.3	169.9 ± 12.0	204.2 ± 14.8	215.8 ± 11.3	75.1 ± 5.0	152.8 ± 10.9	218.3 ± 17.5	229.9 ± 16.6
	NU	94.6 ± 2.3	184.3 ± 7.7	218.3 ± 9.6	215.7 ± 19.9	68.5 ± 5.3	130 ± 11.1	222.9 ± 11.2	221.3 ± 5.7
	NPN	94.6 ± 3.3	179.2 ± 6.2	211.8 ± 6.4	211.9 ± 9.8	69.4 ± 7.4	126.3 ± 16.4	217.8 ± 9.0	217.9 ± 8.3
	p value	ns	ns	ns	ns	ns	ns	ns	ns
Stem girth, mm	Control	15.5 ± 1.3	20.0 ± 0.87	22.2 ± 2.0	22.3 ± 0.69^b^	14.9 ± 1.9	26.2 ± 1.6	26.0 ± 0.82^b^	27.1 ± 0.78^c^
	Urea	16.0 ± 0.88	19.9 ± 1.1	23.8 ± 0.43	23.1 ± 0.68^b^	14.8 ± 0.94	27.8 ± 0.96	31.7 ± 0.68^a^	30.4 ± 0.76^abc^
	PN	15.5 ± 2.0	20.2 ± 1.2	22.5 ± 2.0	23.4 ± 0.95^b^	17.1 ± 1.3	28.2 ± 1.4	28.6 ± 3.2^ab^	28.1 ± 2.1^bc^
	NU	18.1 ± 1.2	23.3 ± 1.4	26.9 ± 1.6	27.3 ± 1.3^a^	14.0 ± 1.1	26.7 ± 1.6	31.5 ± 0.90^a^	31.4 ± 0.63^ab^
	NPN	16.5 ± 0.71	23.1 ± 1.5	27.4 ± 0.51	26.8 ± 0.28^a^	16.0 ± 1.3	25.3 ± 3.2	33.0 ± 0.53^a^	31.8 ± 0.73^a^
	p value	ns	ns	ns	0.002	ns	ns	0.045	0.04
Chlorophyll, SPAD	Control	25.4 ± 1.6^b^	22.1 ± 0.45^c^	20.8 ± 0.76	25.5 ± 0.83	22.8 ± 1.7	25.1 ± 2.0^c^	25.9 ± 1.6^c^	23.1 ± 2.8^b^
	Urea	26.2 ± 1.4^b^	24.9 ± 3.8^bc^	20.3 ± 3.3	26.5 ± 2.3	23.3 ± 2.1	31.9 ± 1.4^a^	38.5 ± 1.7^a^	38.2 ± 1.6^a^
	PN	25.8 ± 3.8^b^	24.8 ± 2.2^bc^	21.8 ± 2.3	26.6 ± 3.0	23.9 ± 2.5	25.4 ± 1.7^bc^	29.8 ± 4.3^bc^	24.9 ± 5.2^b^
	NU	32.7 ± 0.55^a^	31.3 ± 1.5^ab^	26.6 ± 3.1	33.5 ± 3.6	24.0 ± 1.8	30.5 ± 0.88^ab^	37.1 ± 2.58^ab^	36.1 ± 1.3^a^
	NPN	33.4 ± 1.2^a^	34.2 ± 1.5^a^	27.2 ± 1.4	34.3 ± 2.2	21.1 ± 2.2	31.1 ± 2.3^a^	35.2 ± 2.4^ab^	35.9 ± 2.4^a^
	p value	0.024	0.007	ns	ns	ns	0.032	0.022	0.006
LA, cm^2^	Control	309.0 ± 35.6	615.0 ± 51.8	616.0 ± 43.1	560.0 ± 47.9	217.0 ± 28.7	672.0 ± 46.6	849.0 ± 219.2	905.0 ± 193.1
	Urea	325.0 ± 15.9	657.0 ± 10.1	613.0 ± 20.5	801.0 ± 172.3	220.0 ± 21.1	702.0 ± 19.3	983.0 ± 174.2	796.0 ± 30.7
	PN	317.0 ± 33.0	634.0 ± 46.4	609.0 ± 34.4	611.0 ± 48.6	219.0 ± 27.1	704.0 ± 60.4	728.0 ± 64.2	726.0 ± 53.4
	NU	353.0 ± 16.1	708.0 ± 14.0	702.0 ± 13.9	686.0 ± 22.9	185.0 ± 25.8	722.0 ± 44.8	748.0 ± 28.7	763.0 ± 16.7
	NPN	352.0 ± 17.8	718.0 ± 18.5	686.0 ± 24.1	661.0 ± 34.3	201.0 ± 37.5	573.0 ± 122.6	773.0 ± 27.2	798.0 ± 23.0
	p value	ns	ns	ns	ns	ns	ns	ns	ns

DAP, days after planting; control, unfertilized; PN, plasma nitrogen; NU, nano urea; NPN, nano plasma nitrogen; LA, leaf area; ns, not significant. Within the same column and per parameter, means (± SE) followed by the same letters are not significantly different for α = 0.05.

In the first screenhouse, the increase in stem girth owing to fertilizer application was not significant at any crop growth stage. However, the stem girth for plasma-based applications peaked at 42 DAP, whereas that for urea-based fertilizers peaked between 56 and 70 DAP (**Table 2**). In the second screenhouse, from 42 to 84 DAP, nano-fertilizer application resulted in a significant increase in stem girth during each period. At 56–84 DAP, NPN treatment resulted in a significant increase in girth compared with the other treatments. In the 2024A season field trials, the nano-fertilizer treatments increased maize stem diameter, with a significant effect observed at 90 DAP (**Table 3**). Although maize plants treated with nano-fertilizer exhibited earlier peaks at the flowering stage, their stem girth increased significantly at the silking stage, whereas urea, PN, and control treatments peaked later at 90 days after planting (DAP). In the 2024B season, at 70 and 90 DAP, the plots treated with nano-fertilizer demonstrated significant increases in stem girth compared to the control and PN treatments. Although the stem girth for the PN treatment was larger than that for the nano-fertilizer during the early to late vegetative stages in the 2024B season, it peaked at silking, similar to the control. In contrast, urea and nano-fertilizer peaked earlier during the flowering stage.

In the initial screenhouse experiment at 70 days after planting (DAP), NPN application significantly increased the SPAD chlorophyll value compared to the control, urea, and PN treatments (**Table 2**). Furthermore, while the chlorophyll SPAD of maize plants from plots treated with nano-fertilizer reached its maximum at the tasseling stage, other treatments peaked at the silking stage (84 DAP). In the second screenhouse experiment, the NPN treatment exhibited significantly higher SPAD chlorophyll values than the PN, urea, and control treatments at 42, 56, 70, and 84 DAP. At 70 and 84 DAP, the NU treatment resulted in SPAD values that were 1.3 times higher than those of the PN treatment. During the 2024A season, at 35 DAP (early vegetative stage), the SPAD values from the nano-fertilizer treatments were significantly higher than those from the other treatments. However, at 55 DAP, only the SPAD values from NPN were significantly higher than those from urea, PN, and control (**Table 3**). At 55 DAP, the leaves from the NPN treatments had significantly higher SPAD values than those from the control. Nevertheless, the SPAD values across the growth cycle exhibited a parabolic trend, peaking at 70 DAP for all treatments in the 2024A season. In the 2024B season, the SPAD values followed a sigmoidal growth curve. However, at 55 DAP (late vegetative stage), 70 DAP (tasseling), and 90 DAP (silking), the increases in SPAD values from plants treated with urea, NPN, and NU, ranging from 2.6-22.7%, 9.4-48.7%, and 6.4-65.4%, respectively, were significantly higher than those from the control and PN treatments at the same DAP.

There was no significant effect of treatment on leaf area (LA) at any crop growth stage in either the screenhouse or on-station field trials (**Tables 2**, **3**). In the first screenhouse experiment, despite the sigmoid trend in LA, the fertilized plots, particularly those treated with NU and NPN, exhibited LA levels greater than those of the control (**Table 2**). In the second screenhouse experiment, the NU, control, and NPN treatments resulted in plants with larger LA than those treated with urea and PN, respectively. Nonetheless, LA peaked at 84 DAP in all treatments in the screenhouse. During the 2024A season, all treatments peaked at 55 DAP, with the nano-fertilizer showing the greatest increase in LA (**Table 3**). From 35 to 55 DAP during the 2024B season, plants in NPN-treated plots had the lowest LA; from 70 to 90 DAP, plants in PN- and NU-treated plots had the lowest LA, although all treatments reached their peak at 90 DAP.

### 3.2 Effects of urea, PN, and their nano-fertilizer forms on maize yield parameters

The fertilizer treatments did not significantly affect maize yield parameters, except for the harvest index (HI) in the first screenhouse (**Table 4**). The number of kernel rows per maize ear was 2-25.5% higher for nano-fertilizer than for other fertilizer treatments in the screenhouse experiments. Nevertheless, the highest kernel rows was recorded when NPN was applied to the soil. In the 2024A on-station trials, there were negligible increases in kernel rows with NU compared to other fertilizer treatments. However, the use of NPN resulted in 1.8-5.6 more kernel rows than urea, PN, and unfertilized treatments. During the 2024B season, the kernel rows of maize fertilized with the nano-fertilizer decreased by 17.0-24.9% compared to other fertilizers. However, the fewest kernel rows were observed in maize fertilized with NU compared to NPN fertilization.

**Table 4:** Comparative effects of urea, PN, and their nano-fertilizers forms on maize yield parameters.

Fertilizer	Kernel rows	First screenhouse		HI	Kernel rows	Second screenhouse		HI
		Grains/kernel row	100 seed, g			Grains/kernel row	100 seed, g	
Control	9.8 ± 0.25	10.0 ± 0.0	26.2 ± 7.7	0.27 ± 0.006^b^	9.8 ± 1.1	10.0 ± 0.0	21.5 ± 0.91	0.15 ± 0.02
Urea	10.0 ± 0.0	11.0 ± 0.58	29.4 ± 2.1	0.32 ± 0.008^a^	9.5 ± 0.9	11.0 ± 0.58	28.7 ± 4.6	0.15 ± 0.03
PN	9.8 ± 0.25	9.0 ± 1.0	35.3 ± 7.8	0.30 ± 0.0^a^	9.5 ± 1.9	9.0 ± 1.0	28.1 ± 3.4	0.15 ± 0.02
NU	10.0 ± 0.82	10.5 ± 1.3	30.2 ± 6.7	0.31 ± 0.004^a^	10.0 ± 0.58	10.5 ± 1.3	35.3 ± 5.5	0.12 ± 0.02
NPN	10.0 ± 0.82	11.5 ± 1.9	34.4 ± 1.8	0.32 ± 0.009^a^	10.8 ± 0.75	11.5 ± 1.9	34.0 ± 6.0	0.14 ± 0.01
p value	ns	ns	ns	0.001	ns	ns	ns	ns
		2024A season				2024B season		
Control	12.9 ± 0.47	30.0 ± 0.64	33.4 ± 0.42	0.30 ± 0.01	13.0 ± 0.20	38.7 ± 0.86	34.9 ± 1.1	0.38 ± 0.01
Urea	13.2 ± 0.23	33.8 ± 0.92	34.9 ± 0.95	0.31 ± 0.01	13.3 ± 0.21	37.3 ± 0.66	37.6 ± 1.0	0.44 ± 0.01
PN	13.4 ± 0.27	33.1 ± 1.9	35.8 ± 1.2	0.29 ± 0.01	13.1 ± 0.25	35.2 ± 1.9	35.6 ± 2.0	0.34 ± 0.01
NU	13.3 ± 0.16	35.8 ± 1.4	34.8 ± 1.2	0.31 ± 0.01	13.3 ± 0.10	37.0 ± 0.70	37.9 ± 0.34	0.36 ± 0.01
NPN	12.7 ± 0.28	35.6 ± 1.6	37.7 ± 1.1	0.28 ± 0.01	13.7 ± 0.13	36.5 ± 1.2	37.4 ± 1.2	0.36 ± 0.01
p value	ns	ns	ns	ns	ns	ns	ns	ns

Control, unfertilized; PN, plasma nitrogen; NU, nano urea; NPN, nano plasma nitrogen; HI, Harvest Index; ns, not significant. In the same growing period, means (± SE) followed by the same letters are not significantly different at p ≤ 0.05.

In the screenhouse, the application of NU led to increases of 5.0% and 16.7% in the number of kernel per row compared with the control and PN, respectively (**Table 4**). However, the number of grains per kernel row decreased by 4.5% with NU compared to urea. In the screenhouse, the number of grains per row with NPN was 1.1–1.3 times higher than that with the control, urea, and PN treatments. However, NPN application in the screenhouse resulted in a higher number of grains per kernel row than NU. In the on-station field trial during the 2024A season, applying NU or NPN resulted in 2–6 additional grains per kernel row compared to the other treatments. However, in the 2024B season, the grains per kernel row of maize treated with the nano-fertilizer decreased by 0.8-5.7% compared to the control and urea fertilizer. In contrast, the grains per kernel row from the NPN and NU treatments increased by 3.7% and 5.1%, respectively, relative to the 35.2 grains per kernel row from the PN application.

In the initial screenhouse experiment, the 100-seed weight increased by 2.7-15.3% and 17.0-31.3% with the application of NU and NPN, respectively, compared to the control and urea treatments (**Table 4**). However, the seed weight was reduced by 2.6-14.5% relative to the PN treatment owing to the use of nano-fertilizer. In the second screenhouse experiment, the application of nano-fertilizer resulted in an additional yield of 5.3-13.8 g compared to the control, urea, and PN treatments. During the 2024A season, the application of NU increased seed weight by 4.2% compared to the control, although it was reduced by 0.3% and 2.8% relative to urea and PN, respectively. Conversely, the application of NPN in the 2024A season resulted in seed weights that were 5.3%, 8.0%, and 12.9% higher than those of maize treated with PN, urea, and no fertilizer, respectively. Similarly, in the 2024B season, the use of nano-fertilizer increased seed weight by 5.1-8.6% compared to the control. However, the NU seed weight was 0.80% higher than that of PN, whereas the NPN seed weight decreased by 0.5% compared to that of PN seed weight.

In the first screenhouse experiment, the application of fertilizers significantly increased the maize harvest index (HI) by 11.1-18.5 times compared to the control (**Table 4**). Furthermore, the HI from NU was 3.3-14.8% and 6.7-18.5% higher than that of maize without fertilizer and PN, respectively. The application of NU resulted in a 3.1% decrease in maize HI compared to urea fertilizer. Similarly, in the second screenhouse, NU and NPN reduced HI by 20.0% and 16.7%, respectively, compared with the control, urea, and PN treatments in the second screenhouse. Conversely, in the 2024A season field trials, the HI from NU was 3.3% and 8.9% higher than that from maize without fertilizer and PN, respectively. However, in the same season, maize treated with NPN exhibited a 3.5-9.7% reduction in HI compared to the control, urea, and PN. In the 2024B season, the nano-fertilizer caused a 5.3-18.2% reduction in maize HI compared to the control and urea treatments. In contrast, the nano-fertilizer increased the maize HI by 5.9% compared to PN.

### 3.3 Effects of urea, PN, and their nano-fertilizer forms on maize grain yield

**Figure 5:**
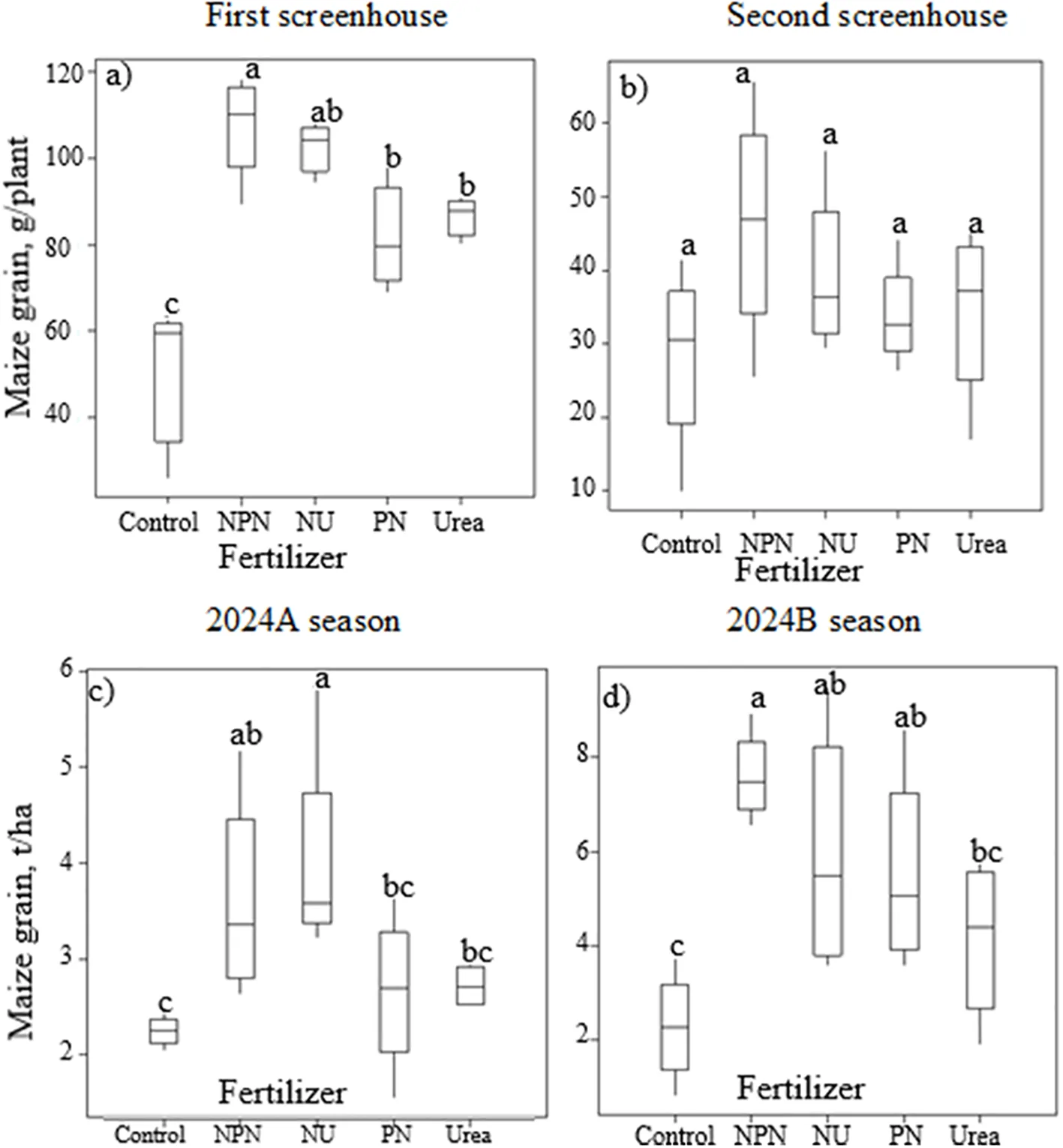
Effects of fertilizers on maize grain yields in screenhouse **(a, b)** and on-station field trials **(c, d)**.

Significant variations in maize grain yield were observed across different fertilizer treatments, with the exception of the second screenhouse (screenhouse one, p < 0.001; screenhouse two, p < 0.366; on-station trial: 2024A season, p < 0.043; 2024B season, p < 0.014). The application of nano-fertilizer resulted in higher maize grain yields than those of plots treated with urea, PN, or control (no fertilizer). In the first screenhouse, the highest maize yield of 107.1 g per plant from pots treated with NPN was significantly greater than those treated with PN, urea, and the control, by factors of 1.66, 1.74, and 2.2, respectively (**Figure 5**). In the second screenhouse, the yield was maximized with the nano-fertilizer, exceeding those with the Urea or PN fertilizer and the control by 5.5-12.3 g and 11.5-18.2 g, respectively. During the 2024A season, the 4.05 t ha^-1^ maize grain yield from plots treated with NU was significantly higher than that from the PN, urea, and control treatments by 52.8%, 48.9%, and 80.8%, respectively. Meanwhile, the 3.63 tons from plots with NPN surpassed PN and urea treatments by 37.0% and 33.5%, respectively, while the 2.24 tons from the control was significantly lower than that from the NPN treatment. In the 2024B season, the highest grain yield with NPN was 36.4% above that of PN and significantly higher than that of urea and the control by 85.2% and 235.2%, respectively. Although NU produced 6.01 t ha^-1^, which was 7.7% and 46.2% higher than that of PN and urea, respectively, this yield was also significantly higher than the 2.27 t ha^-1^ yield from the control.

### 3.4 Effects of urea, PN, and their nano forms on NUE

The nitrogen recovery efficiency (RE) of maize cultivated in either a screenhouse or an open field was influenced by the type of fertilizer used, with significant differences observed only in the 2024B season (**Table 5**). The highest RE was recorded with NPN application, followed by NU application. In the first screenhouse, the RE for maize treated with NU and NPN was 60.2% and 83.4%, respectively, which were 1.1-1.7 times higher than those achieved with PN or urea application. Conversely, in the second screenhouse, the RE of maize treated with the nano-fertilizer was 1.3 to 3.5 times higher than that of maize treated with PN or urea.

**Table 5:** Comparative effects of urea and PN and their nano-fertilizer forms on nutrient use efficiency in maize.

Fertilizer	Screenhouse one					Screenhouse two				
	RE, %	APE, kg kg^-1^ N	PE, kg kg^-1^ N	AE, kg kg^-1^ N	NHI, %	RE, %	APE, kg kg^-1^ N	PE, kg kg^-1^ N	AE, kg kg^-1^ N	NHI, %
Control	na	na	na	na	31.8 ± 3.8^b^	na	na	na	na	17.2 ± 2.2
Urea	56.9 ± 9.3	14.8 ± 3.6	53.5 ± 15.6	23.0 ± 15.2	32.3 ± 0.81^b^	19.2 ± 10.4	1.4 ± 1.5c	46.9 ± 14.9	3.6 ± 3.3	14.3 ± 1.8
PN	49.8 ± 16.1	20.5 ± 3.9	45.0 ± 20.6	22.7 ± 13.8	37.4 ± 2.0^a^	10.0 ± 9.3	17.3 ± 2.1a	94.5 ± 29.5	8.3 ± 9.8	16.7 ± 2.7
NU	60.2 ± 8.9	21.8 ± 2.1	70.8 ± 10.4	35.8 ± 15.9	29.7 ± 0.60^b^	35.4 ± 2.8	4.0 ± 2.3c	44.2 ± 9.3	11.5 ± 4.5	12.9 ± 1.8
NPN	83.4 ± 10.87	16.0 ± 1.6	44.7 ± 6.8	38.4 ± 4.2	30.8 ± 0.82^b^	24.7 ± 6.8	7.6 ± 2.1b	58.2 ± 5.7	12.6 ± 4.6	15.6 ± 1.3
p value	0.317	0.056	0.50	0.168	0.022	0.255	0.001	0.137	0.488	0.546
LSD_0.05_	44.78	5.604	42.99	19.17	4.115	27.28	3.319	48.35	15.06	5.932
	2024A season					2024B season				
Control	na	na	na	na	35.8 ± 1.7^a^	na	na	na	na	37.2 ± 0.99
Urea	15.8 ± 3.0	129 ± 83.5	113.8 ± 34.4^a^	7.2 ± 1.6	35.1 ± 1.6^a^	28.1 ± 15.2^b^	54.2 ± 38.8	55.3 ± 12.4	27.9 ± 10.5	43.8 ± 9.4
PN	37.1 ± 10.5	14.0 ± 4.0	52.8 ± 11.2^b^	17.7 ± 6.4	29.2 ± 1.3^ab^	53.3 ± 10.0^c^	16.7 ± 5.1	50.6 ± 7.7	50.1 ± 14.5	33.7 ± 3.3
NU	48.3 ± 16.7	16.0 ± 5.9	48.2 ± 17.3^b^	27.4 ± 8.0	25.2 ± 2.9^bc^	84.0 ± 16.7^a^	10.8 ± 4.6	31.6 ± 12.9	56.6 ± 29.5	35.7 ± 0.66
NPN	51.2 ± 16.4	6.0 ± 1.9	23.9 ± 3.5^b^	21.0 ± 7.9	20.3 ± 1.4^c^	75.1 ± 10.0^a^	20.2 ± 2.1	57.9 ± 8.1	80.9 ± 9.2	36.1 ± 0.67
p value	0.073	0.211	0.039	0.077	0.003	0.014	0.416	0.359	0.196	0.432
LSD_0.05_	28.39	138.1	59.27	15.1	6.389	7.307	61.01	34.63	50.31	14.12

Control, unfertilized; PN, plasma nitrogen; NU, nano urea; NPN, nano plasma nitrogen; RE, recovery efficiency; APE, agrophysiological efficiency; PE, physiological efficiency; AE, agronomic efficiency; NHI, nitrogen harvest index; na, not applicable; LSD, least significant difference. In the same growing period, means (± standard error) followed by the same letters are not significantly different at p ≤ 0.05.

During the 2024A season, the recoverable efficiency (RE) of the nano-fertilizer exceeded that of maize treated with urea or PN by 11.2–35.4%. In the 2024B season, the highest RE was 84%, achieved with NU, significantly surpassing that of NPN, urea, and PN by 7.3%, 199.0%, and 57.6%, respectively. However, the RE of 75.1% for NPN was notably higher than those for urea and PN by factors of 2.7 and 1.4, respectively. Nonetheless, in this study, the RE of nitrogen from PN was 1.9 times greater than that from urea.

The physiological efficiency (PE) of maize grown with different fertilizer types varied significantly only during the on-station trials of the 2024A season (**Table 5**). The highest PE (70.8 kg kg^-1^ N) in the first screenhouse, achieved with NU, was 32.3% and 57.3% higher than that of N applied as urea and PN, respectively. Conversely, the PE of maize grown with NPN was reduced by 8.8 kg kg^-1^ N and 0.3 kg kg^-1^ N compared to urea and PN, respectively. In the second screenhouse experiment, the PE of maize with NU (58.2 kg kg^-1^ N) was 5.8% and 53.2% lower than that with urea and PN, respectively. In the 2024A field trials, urea applied to maize resulted in a significantly higher PE than that of the other fertilizers. The PE of maize with NU and NPN declined by 65.6 and 89.9 kg kg^-1^ N, respectively, compared with that of urea. However, the PE of maize with NU and NPN was 4.6 and 28.9 kg kg-1 N, respectively, lower than that of PN. In the 2024B season, the PE of NU decreased by 23.7 and 19.0 kg kg^-1^ N compared to urea and PN, respectively. Nonetheless, the PE of maize with NPN was higher by 2.6 kg kg^-1^ N with urea and 7.3 kg kg^-1^ N with PN.

The agro-physiological efficiency (APE) of maize cultivated with various fertilizers showed significant variation only in the second screenhouse experiment (**Table 5**). While nano-fertilizers increased APE by 1.1-1.5 times compared to urea in the first screenhouse, NU increased APE by 1.3 kg kg^-1^ compared to PN, whereas NPN led to a 22.0% decline compared to PN. In the second screenhouse, PN produced a significantly higher APE than the other fertilizer treatments. Furthermore, the APE of NPN was significantly higher than that of NU and urea by 3.6 and 6.2 kg kg^-1^ N absorbed by the plant, respectively. During the 2024A and 2024B seasons, the APE of maize treated with nano-fertilizer decreased by 87.6-95.5% and 62.7-80.1%, respectively, compared to urea fertilizer. However, the APE of NU increased by 2.0 kg kg^-1^, whereas that of NPN decreased by 8.0 kg kg^-1^ compared with PN in 2024A, respectively. In the 2024B season, the APE of NPN decreased by 35.3%, whereas the APE of NU increased by 21.0% compared with that of PN.

There was no significant increase in the agronomic efficiency (AE) of maize grown with nano-fertilizers compared to that grown with urea and PN (**Table 5**). The highest AE was observed with NPN, except during the 2024A season, when the values were low, with the highest at 27.4 kg kg^-1^ of applied N from the NU fertilizer. Maize treated with nano-fertilizer in a screenhouse demonstrated AE 55.6-250% higher than that treated with urea. However, the increase in AE with nano-fertilizer ranged from 36.6% to 15.7% above that of the PN treatment group. Similarly, in on-station trials during both the 2024A and 2024B seasons, the AE of maize in plots treated with nano-fertilizer was 102.9-280.6% higher than that with urea. Compared to PN, the AE of the nano-fertilizer was 1.2-1.6 times higher.

Significant alterations in the nitrogen harvest index (NHI) were observed exclusively in the first screenhouse during the 2024A season (**Table 5**). Within this screenhouse, the NHI of maize treated with non-fertilizer experienced a substantial decrease of 17.5-20.6 units from the 37.4% recorded with PN. Conversely, in the second screenhouse, maize treated with nano-fertilizer exhibited a reduction in NHI by 1.1-7.7 points compared to maize treated with other fertilizer amendments. Notably, the NHI of maize treated with NPN was 1.1 times greater than that of maize treated with urea in the second screenhouse. During the on-station trial in the 2024A season, maize from the control or urea treatments demonstrated significantly higher NHI than that from the nano-fertilizer treatments. Furthermore, the NPN treatment exhibited an NHI that was 8.9 units lower than that of the PN treatment. The NHI of maize from plots treated with nano-fertilizer was 4-14.8 points lower than that of maize treated with urea and PN. In the 2024B season, maize treated with NU had NHI values 7.7-8.1 points lower than those of urea-treated maize. However, the NHI of nano-fertilizer was 2-2.4 units higher than that of PN treatment.

## 4 Discussion

### 4.1 Effects of urea, PN, and their nano-fertilizer forms on maize growth in screenhouse and field trials

The results indicated that soil fertilization significantly improved maize growth characteristics, consistent with most studies (see Kyomuhendo et al., 2018) reporting 80% nitrogen (N) deficiency in Ugandan soils, which limits crop growth responses and provides recommendations for the appropriate use of fertilizers. The improved plant height, stem girth, chlorophyll, and leaf area (LA) observed in plots treated with nano-fertilizers compared to those treated with plasma nitrogen (PN) or urea could be attributed to the better timing of N availability (Davies et al., 2020). The slow release of N from nano-fertilizers could sustain synchrony in nutrient supply, leading to increased plant height, stem girth, chlorophyll content, and LA. Dey and Sadhukhan (2024) and Sadiq et al. (2021) reported similar plant growth results with prolonged N availability.

In contrast, the shorter plants associated with fertilizer use (**Tables 2** and **3**) may be related to an excessive N supply (Ma et al., 2025). Specifically, the relationship between nitrogen levels and plant height is crucial; excessive nitrogen fertilizer promotes vegetative growth and can reduce stem elongation, resulting in shorter plants (Sharifi and Namvar, 2016). Li et al. (2024) reported reduced stem internode elongation associated with enhanced meristematic activity before flowering (**Tables 2**, **3**), thereby increasing sink strength and the number of grains per panicle. Nevertheless, the sustained nutrient release ability of nano-fertilizers could also have increased the nitrogen supply capacity of the soil for crops, causing larger leaf areas and enhanced chlorophyll SPAD values (**Tables 2** and **3**), which could improve light energy utilization (Wang et al., 2021) and increase photosynthesis (Szulc et al., 2021; Chen et al., 2023). The SPAD index, which measures chlorophyll content, is a reliable indicator of plant health and stress levels (Shivashankar et al., 2025). The promotion of improvements in the photosynthetic rate and crop productivity using NU or NPN was similarly reported for nano-fertilizers by Liu et al. (2023b).

However, an earlier study by Kenawy et al. (2020) showed that flag leaf photosynthetic activity and stem assimilate transfer to the grain are fundamental determinants of the grain-filling ratio and grain mass in cereals. A decrease in stem girth from the late vegetative to the silking stage could result from an imbalance in N supply relative to crop demand (**Tables 2** and **3**) and antagonistic interactions with other nutrients (Kurdestani et al., 2024; Enock et al., 2026), which requires further study. Specifically, understanding how N supply varies across growth stages and soil conditions when using nanofertilizers is essential. A similar study (Quansah, 2010) attributed the shrinkage in stem girth to the translocation of photosynthates during grain formation. According to Li et al. (2024), reduced stem girth is also associated with a decrease in the number of large vascular bundles and in the cross-sectional area of the xylem and phloem, which are important for non-structured carbohydrate and nitrogen translocation. These findings are consistent with those of Ning et al. (2017) and Winnie et al. (2022), who reported a significant decrease in N concentration in maize stover and a greater increase in N concentration in maize grain under both low and high-N inputs. These results suggest specific N supply levels for different growth stages under varying soil conditions when using nano-fertilizers.

### 4.2 Effects of urea, PN, and their nano-fertilizer forms on maize yield and nitrogen use efficiency

The non-significant increase in yield components (**Table 4**) may be attributed to the recommended nitrogen (N) fertilizer rate for conventional fertilizer being adequate to achieve the crop’s maximum yield (Liu et al., 2023b). The stimulation of yield components by nano-urea (NU) and NPN aligns with the findings of Sadiq et al. (2021), who reported the variable effects of nano-fertilizers on growth and yield parameters. Conversely, Kenawy et al. (2020) observed that N fertilization enhanced growth traits and influenced yield parameters, including the kernel rows and seed weight. These variations likely arise from biotic and abiotic factors, including genetic and environmental influences such as temperature (Szulc et al., 2021; Liu et al., 2023a). These results suggest that the combined response of yield attributes to the applied N resulted in a synergistic increase in the grain yield (Kyebogola et al., 2021; Enock et al., 2026; **Figure 5**). Further analysis using structural equation modeling or correlation analysis could elucidate the combined impact of each parameter on grain yield. Notably, the 33.5-85.2% improvement in grain yield from NPN compared with chemical fertilizer exceeded the 30% reported by Mejias et al. (2021) for nano-fertilizers. Thus, the controlled release of N from the nano-fertilizer enabled nutrient availability to the plant when required for its physiological yield parameters, thereby supporting a higher grain yield (**Figure 5**). Moreover, NPN and NU produced higher grain yields than the plots treated with PN, urea, or unamended soil, underscoring the importance of nanocellulose encapsulation in controlling nitrogen release to support crop development. Mechanistically, the nanocellulose surface and N-containing compounds in nano-fertilizers induce functional groups such as -NH_2_ and -CO-. These groups interact via hydrogen bonding. This interaction creates a more compact polymeric network (Kassem et al., 2021). As a result, the nano-fertilizers exhibited enhanced N retention and controlled slow-release behavior. The functional groups also facilitated N adsorption. In addition, the matrix expands with water diffusion while the pores in the matrix shrink or collapse. This is due to the stronger intermolecular forces and weaker intramolecular forces in the polymer chain. Thus, the matrix becomes more compact. Furthermore, the fibrous and porous network of nanocellulose creates a tortuous diffusion path for the solvent. This slows the release of N into the soil, allowing for sustained plant nutrient uptake and adequacy during critical periods, reducing losses (Kyebogola et al., 2024), and improving maize growth and yield (**Tables 2**–**4**; **Figure 5**). Integrating agronomic, social, and economic factors into the targeting of nano-fertilizers for on-farm maize production is important for prioritizing NU and NPN under all field conditions. Nevertheless, the results indicate that NPN can replace urea and PN to achieve yields comparable to NU fertilizers, a finding that can be further validated through on-farm evaluations across various agroecologies. These observations are consistent with those of Kenawy et al. (2020) and Wang et al. (2021), who demonstrated that nano-fertilizers can enhance cereal crop growth and yield. Additional benefits of nano-fertilizers, including increased resistance to high soil temperatures, reduced water stress, and reduced leaching, have been documented (Curtis et al., 2020; Kenawy et al., 2020). These findings suggest that NPN application during low-rainfall periods (**Figure 2**) further enhances maize yields (**Figure 5**). Thus, future studies should confirm the climate-based efficacy of NPN fertilizers.

Remarkably, the enhanced Nitrogen Recovery Efficiency (RE) observed in maize plants from plots treated with nano-fertilizer, as compared to those treated with PN or urea, indicates more efficient nutrient uptake and reduced waste. Similar findings for nano-fertilizer treatments have been documented for split applications of N fertilizer (Trenkel, 2010). The RE achieved with nano-fertilizers surpassed the commonly reported global average of approximately 30-50% for conventional fertilizers (Beesigamukama et al., 2020; Davies et al., 2020; Rahman et al., 2021; Govindasamy et al., 2023). However, the field-based RE with NU and NPN is comparable to the 71% reported by Elsworth and Paley (2009) for slow-release fertilizer, but lower than the 130% reported by Nezami et al. (2021) for nano-fertilizer. This variability in performance suggests the need for improvements in the nanoformulation process. Alternatively, Aslam et al. (2026) observed optimized wheat productivity and NUE when 75% of the recommended N rate was applied, thereby reducing the fertilizer use. Furthermore, the relatively stable RE with nano-fertilizers across maize-growing periods indicates its potential to withstand varying weather and soil conditions (**Figures 2**–**4**; **Table 1**), with the possibility of further increasing with appropriate application timing (Davies et al., 2020). According to Rahman et al. (2021), this nutritional enhancement from nano-fertilizers has been considered a mechanism against plant pathogens, as pests and diseases cause 20-40% yield reduction and economic losses, threatening food safety. Additionally, the high RE from nano-fertilizers has the potential to reduce environmental contamination by fertilizers, suggesting that low and seasonal variations in RE underscore the importance of adapting PN and urea fertilizer strategies to specific nanoencapsulations, split applications, and cropping seasons. The RE obtained with nano-fertilizer, similar to the 80% reported for the wheat-maize system in Europe and America (Liu et al., 2010), suggests a potential strategy for optimal N fertilizer management in agricultural practices through nanoencapsulation.

While nitrogen (N) uptake in maize from plots treated with nano-fertilizer was substantial, it did not necessarily translate into optimal Physiological Efficiency (PE), underscoring the importance of appropriate application rates, as previously discussed. High N bioavailability and luxury uptake lead to reduced PE (Winnie et al., 2022), thereby challenging the support for environmental sustainability in agriculture (Ali et al., 2025). Conversely, the limited N uptake from PN and urea improved PE, likely due to an optimal nutrient balance, including phosphorus (P) and potassium (K) (Kurdestani et al., 2024). Consistent with this, the PE values obtained predominantly fell within the 53.3-86.6 kg kg^-1^ range reported by Godebo et al. (2021) for irrigated wheat under varying K levels. Understanding the slow, gradual, and sustained release of different N forms through nanoencapsulation, which helps control soil conditions such as pH to improve the availability of essential nutrients (N, P, K, Ca, Mg, and S), would enable an adequate account of the supply to plant roots and their utilization (Aslam et al., 2026). The high Agrophysiological Efficiency (APE) of maize under screenhouse conditions when treated with nano-fertilizer or PN reflects the plant’s capacity to convert the applied N into an economic yield (**Table 5**). Similarly, Liu et al. (2010) reported an APE for maize ranging from 5.5 to 87.8 kg grain kg^-1^ N. Furthermore, Randhawa et al. (2021) attributed the differences between PE and APE to crop requirements being met with a lower N uptake. Conversely, the efficient utilization of low-N absorbed from urea and PN for grain formation further underscores the need to optimize NPN and NU application rates in maize.

Nevertheless, the Agronomic Efficiency (AE) of maize treated with nano-fertilizer was superior, indicating an adequate nitrogen supply for grain yields within the AE range for fertilizer applied between the silking and maturity stages (Ning et al., 2017). This finding further underscores the necessity of appropriately timing nano-fertilizer application to enhance AE (Davies et al., 2020). Similarly, the AE of maize from plots treated with nano-fertilizer fell within the 30.3-83.6 kg kg^-1^ range reported by Randhawa et al. (2021) for integrated nutrient management of cereals. These values highlight the significance of understanding agricultural practices and the effects of climate variability (**Figures 2**–**4**; **Table 1**), including rainfall distribution, on the economy of the region. Such understanding could enhance the AE of maize with NU or NPN applications (**Table 5**) beyond the 31.0 kg kg^-1^ reported for nano-N application by Mejias et al. (2021). The results of this nano-fertilizer study, when compared with those of PN and urea, contradict the findings of Winnie et al. (2022), who demonstrated that increasing N uptake reduces AE to optimal values of 10–30 kg kg^-1^. These results suggest that increased Recovery Efficiency (RE) and Physiological Efficiency (PE) are more closely associated with higher AE for both nano and conventional fertilizers. In contrast, the higher Nitrogen Harvest Index (NHI) values for maize from plots treated with urea or PN, compared to nano-fertilizer treatments, emphasize the importance of breeding efforts to enhance nutritional quality, as RE is augmented by sustained nano-fertilizer N release. However, the NHI values obtained were significantly lower than those reported by Folina et al. (2021) and Li et al. (2024), likely due to differences in rainfall and temperature between the silking and harvesting periods (**Figures 2**–**4**), suggesting potential effects on the crop quality.

## 5 Limitations of the study

This study is limited by its two-season, single-site design for both screenhouse and on-station trials, which restricts generalizability across environments. In addition, the study requires an on-farm study to bridge the gap between controlled screenhouse or research-station trials and real-world agriculture. Furthermore, the 1% N in PN is too low compared with advancements in plasma technology that use a series of reactors to produce high concentrations of N (Kyebogola et al., 2024), which is one of the challenges of the nano-plasma fertilizer (NPN) production process, which increases N concentrations to 15%. Therefore, amid limited labor, it is constraining to apply the required large amount of PN, as well as for storage and transport if not produced on-farm. Moreover, the water volume of PN, which is advantageous for drought control, was not equal across the treatments in this study. In addition, the nanofertilizer was produced at the laboratory scale, and the feasibility and cost-effectiveness of large-scale production require further investigation in the future. In addition, the long-term environmental fate was not evaluated in this trial.

## 6 Conclusions

This study evaluated the performance of Nano Plasma Nitrogen (NPN) through a comparative analysis with nano-urea (NU), urea, Plasma Nitrogen (PN), and the control in maize cultivation. This comparison identified alternative fertilization strategies that enhance maize yield and NUE, thereby providing insights into fertilizer characteristics and promoting environmental sustainability. The findings indicated that different fertilizer types significantly influenced maize growth and yield characteristics throughout all growth stages in both screenhouse and on-station trials. Notably, the nano-fertilizer demonstrated superior performance compared to the control, urea, and PN fertilizers with relative efficacy varying by season. Furthermore, the application of NU and NPN fertilizers resulted in improved maize yield and N use efficiency. The high nitrogen recovery efficiency (RE), agrophysiological efficiency (APE), agronomic efficiency (AE), and nitrogen harvest index (NHI) observed with a single application of nano-fertilizers at planting suggest the potential to reduce split applications, minimize waste, and enhance grain nutritional quality. This practice contributes to the sustainability of agriculture. Additionally, the performance stability of NU and NPN indicates their applicability as slow-release fertilizers across diverse environments, highlighting the need to nanoencapsulate urea or plasma-nitrogen-based fertilizers to avoid continuous application and mitigate the associated risks. Consequently, like NU, NPN can be adopted as an environmentally safe and sustainable fertilizer option for maize production, depending on weather conditions, thereby inspiring innovative climate-smart approaches to developing slow-release fertilizers. Thus, utilizing a sustainable circular production process for NPN will decrease the overreliance on mineral nitrogen derived from the Haber-Bosch process.

## Data Availability

The raw data supporting the conclusions of this article will be made available by the authors, without undue reservation.
