# Supplementary material for: Exploring slow-release plasma nitrogen nano-fertilizer for improved maize production and nutrient use efficiency
Source: Front Plant Sci. 2026 Sep 10;17:1883384. doi: 10.3389/fpls.2026.1883384 (PMC13600992; doi:10.3389/fpls.2026.1883384)
Supplement: Supplementary file 1 [file Table1.docx]

Properties of nano-fertilizers.

| Nano fertilizer | DOC (%) | R^2^ | X^2^ | FWHM |
| --- | --- | --- | --- | --- |
| NU | 74.6 | 66.8 | 30.6 | 49.4 |
| NPN | 64.4 | 57.1 | 14.3 | 8 |

*DOC =degree of crystallinity, R^2^=regression, X^2^=Chi-square, FWHM=full width half maximum.
